# Effects of potassium fertilizer application on *Qiongzhuea tumidinod* shoots nutritional quality and soil nutrients

**DOI:** 10.3389/fpls.2025.1686259

**Published:** 2025-12-10

**Authors:** Weidong Li, Wenyuan Dong, Huan Zhong, Tingxuan Fu, Jixia Duan, Jianye Sun, Yiyuan Wu

**Affiliations:** 1 College of forestry, Southwest Forestry University, Kunming, China; 2 Yunnan Institute of Biodiversity, Southwest Forestry University, Kunming, China

**Keywords:** *Qiongzhuea tumidinoda*, potash fertilizer, bamboo shoot quality, soil nutrient, analysis of relationship

## Abstract

**Introduction:**

The effects of potassium fertilization on the quality of Qiongzhuea tumidinoda shoots and soil nutrients were investigated by using a completely randomized block design with no fertilization as the blank control group (CK). In order to provide aiming to establish a theoretical foundation for optimizing cultivation practices and fertilization strategies in Q. tumidinoda forests.

**Methods:**

The effects of four different levels of potassium fertilizer application [K1 (75 kg/ha), K2 (150 kg/ha), K3 (225 kg/ha), K4 (300 kg/ha)] on different nutrients (ash, crude fiber, protein, crude fat, soluble sugar, starch) and amino acid content of Q. tumidinoda shoots and soil nutrient content were studied.

**Results:**

The application of potassium fertilizer significantly reduced the ash content of Q. tumidinoda shoots but had no significant effect on crude fiber content. However, it significantly influenced soluble sugar, starch, and protein levels, which exhibited an initial increase followed by a decrease. K3 treatment had the highest soluble sugar content, K2 treatment had the highest starch content, and K3 treatment had the lowest crude fat content. There was no significant difference in the content of essential amino acids between different treatments, but the application of potassium fertilizer significantly affected other amino acid components, especially significantly promoted the accumulation of umami amino acids and sweet amino acids, indicating that the application of potassium fertilizer improved the umami and sweet taste of Q. tumidinoda shoots. With the increase of potassium application rate, the contents of soil organic matter, total nitrogen and total potassium increased first and then decreased, while the content of total phosphorus increased continuously. In the 0~20 cm soil layer, the highest contents of soil organic matter, total nitrogen, total phosphorus, total potassium, hydrolyzed nitrogen, available phosphorus and available potassium were K2, K1, K4, K1, K2, K2 and K1 treatments, respectively. In the 20~40 cm soil layer, the highest contents of soil organic matter, total nitrogen, total phosphorus, total potassium, hydrolyzable nitrogen, available phosphorus and available potassium were K2, K1, K4, K2, K4, K3 and K1 treatments, respectively.

**Discussion:**

Appropriate application of potassium fertilizer can not only improve the nutritional quality of bamboo shoots, but also significantly improve the content of soil organic matter and nitrogen, phosphorus and potassium nutrients. The optimal application amount of potassium fertilizer in this study was K2 treatment, that is, 150 kg/ha.

## 1 Introduction

Reasonable fertilization can increase crop yield, improve soil fertility and economic benefits (Yang et al., 2020). Potassium is one of the three major nutrient elements necessary for plant growth and development (Rmheld and Kirkby, 2010). It has the functions of promoting the activation of various enzymes (Liu et al., 2012; Cui et al., 2011; Lu et al., 2013; Wang et al., 2005), regulating osmotic potential (Krumm et al., 1990) and improving plant stress resistance (Lu et al., 2013; Wang et al., 2014), and can enhance plant photosynthesis and plant stress resistance (Miao and Zhang, 2012). Potassium in soil can be divided into water-soluble potassium, exchangeable potassium, non-exchangeable potassium and mineral potassium according to its form (Bao et al., 2023). The soil is rich in mineral potassium, and the potassium formed by previous ore weathering can meet the needs of agricultural production (Li, 2021). With the rapid development of agriculture and the change of natural conditions in recent years, the content of soil available potassium in most farmlands in China is still at a low level. In recent years, with the continuous introduction and promotion of high-yield varieties, the potassium absorbed and taken away from the soil by crops every year shows an increasing trend, and the potassium in farmland is generally deficient (Gu et al., 2020). There are two main ways of potassium loss from soil: one is the leaching loss caused by poor soil potassium fixation ability and rainfall (Wen et al., 2021); second, part of potassium is taken away with the removal of crop straw (Rmheld and Kirkby, 2010)

Potassium is an essential nutrient element for plants, and it is called the three elements of fertilizer together with nitrogen and phosphorus. The application of potassium fertilizer can increase and stabilize crop yield (Li et al., 2003; Dong et al., 2008) and improve crop quality (Sun et al., 2019). Relevant studies have found that the application of potassium fertilizer can significantly promote the growth and yield of peanut (Long et al., 2023). At the same time, some studies have pointed out that the application of potassium fertilizer in a certain range makes the dry matter accumulation and yield of crops show a positive correlation with the amount of potassium fertilizer, but when the amount of potassium fertilizer is too high, it will significantly inhibit the growth and yield of crops (Du et al., 2017; Xu et al., 2023). Hui Wei et al (Hui et al., 2021). found that rational application of potassium fertilizer can promote the growth and development of quinoa, while excessive potassium fertilizer not only causes the growth of quinoa to be blocked, but also causes potassium fertilizer waste. Zhao Fanghua et al (Zhao and Jiang, 2015) found that under the condition of applying certain potassium fertilizer, tomato yield and quality traits have been improved to a certain extent, indicating that reasonable application of potassium fertilizer can increase tomato yield and improve tomato quality. Trinh et al (Trinh et al., 2023). showed that the application of potassium fertilizer could significantly increase the protein and fat content of peanut. Yan Yusi et al (Yan et al., 2022). pointed out that the application of potassium fertilizer can improve the acidity of grape fruit and increase the content of soluble sugar, vitamin C and other quality indicators. Wang ZhiYing et al (Wang et al., 2025). found that potassium with appropriate concentration could not only promote the absorption and utilization of nitrogen, phosphorus and other nutrients in flue-cured tobacco, but also promote photosynthesis and increase the content of photosynthetic pigments in leaves, thus improving the overall quality of tobacco leaves. Therefore, scientific application of potassium fertilizer can have a positive impact on increasing yield (Tan et al., 2020).

The application of potassium fertilizer can promote the absorption of soil nutrients by bamboo, and significantly increase the contents of crude fiber, amino acid and ash in bamboo shoots, while the contents of crude protein, oxalic acid and amino acid in bamboo shoots are also affected by different fertilization gradients. In previous studies, it was found that the protein and amino acid contents of bamboo shoots in *Q. tumidinoda* forest after fertilization were higher than those in natural *Q. tumidinoda* forest. Liu Shun et al (Liu et al., 2013). studied the effects of potassium fertilizer and balanced fertilization on bamboo forest. In a certain range, the higher the potassium content, the more the bamboo shoots on the day, indicating that potassium fertilizer had a significant effect on the amount of bamboo shoots. Relevant research results show that potassium fertilizer can effectively increase the ground diameter of bamboo shoots and the diameter at breast height of new bamboo. The continuous fertilizer effect of each treatment of balanced fertilization has a promoting effect on the shooting rate, the average daily growth of shoots, and the relative growth of the average DBH of the mother bamboo, but the amount of potassium fertilizer is one of the important factors affecting its growth. Appropriate amount of potassium fertilizer has a promoting effect. Excessive potassium fertilizer will break the balance of N, P and K and inhibit the effect of fertilizer. Zhong DongYang et al (Zhong et al., 2023). screened the formula of liquid microbial fertilizer for Phyllostachys praecox, and found that potassium could effectively improve the number of shoots of mother bamboo, the diameter at breast height and height of new bamboo. Zeng QingNan et al (Zeng et al., 2023). showed that the effect of different solid microbial fertilizer formulations on the growth of Phyllostachys praecox showed that the effect of potassium on the average DBH of new bamboo reached a very significant level.

Bamboo shoots are buds growing on the rhizomes of bamboo, which are mostly harvested after growing to 25 ~ 30 cm. Bamboo shoots are popular because of their crisp, delicious taste, high protein, low fat, rich in amino acids, minerals and dietary fiber (Chen et al., 2023). As a traditional dish in China, bamboo shoots have been eaten and cultivated for more than 2500 years. They are loved by people because of their delicious taste, crisp taste and rich nutrition, and are known as one of the five most popular health foods (Zhang et al., 2021). At present, the research on the quality of bamboo shoots mainly focuses on the effects of nutritional dynamics (Xu et al., 2008; Zhou et al., 2013), storage and packaging methods (Wang et al., 2016, 2006) and management measures (Qiu et al., 2017; Hu et al., 2004) on the nutritional components of bamboo shoots. Among them, a large number of studies have shown that fertilization can significantly affect the yield of bamboo shoots (Hu et al., 2004; Guo et al., 2003) and the content of nutrients such as amino acids, proteins and fats in bamboo shoots (Qiu et al., 2017; Chen et al., 2007). *Qiongzhuea tumidinoda* is a small and medium-sized bamboo plant in the genus *Qiongzhuea* of Poaceae, which is an excellent bamboo species for both shoot and timber (Li et al., 2024). The bamboo shoots of *Q. tumidinoda* are sweet, fresh and tender, crisp, rich in various nutrients, and of high quality. More than 90% of the bamboo shoot products are sold to the international market, which has important edible, economic and ecological value. With the deepening of the construction of the whole industrial chain of Qiongzhuea tumidinoda in Daguan County, the demand for Qiongzhuea tumidinoda shoots and timber resources is increasing. At the same time, the soil in the Qiongzhuea tumidinoda forest is mostly acidic, and the soil is generally weak in fertilizer retention, rich in nitrogen, organic matter, and lack of potassium, which also limits the growth of Qiongzhuea tumidinoda to a certain extent (Xia et al., 2022). In this study, *Q. tumidinoda* forest in Mugan Town, Daguan County, Yunnan Province was taken as the research object. The effects of different potassium fertilizer application rates on the quality of *Q. tumidinoda* shoots and soil nutrient content were discussed. The most suitable potassium fertilizer application rate under the experimental conditions was obtained by comparison, which provided theoretical and practical basis for the production of *Q. tumidinoda* shoots and the sustainable management of bamboo forests.

## 2 Materials and methods

### 2.1 Overview of the study area

The study area is located in Mugan Town, Daguan County, Yunnan Province (103°52′~104°01′ E, 28°02′~28°14′ N), located in northeastern Yunnan, with an altitude of 980-2–263 m; it belongs to the north subtropical monsoon climate type, with an average annual temperature of 11°C and an average annual precipitation of 1–000 mm, mainly concentrated in June-August; the soil is mostly yellow soil and purple soil. There are 16,000 ha forest land in Mugan Town, of which the area of *Q. tumidinoda* forest is the largest. The *Q. tumidinoda* forest in the study area adopts the intensive management mode, and the old bamboos of more than 3 years are harvested every year. The existing bamboos are mainly 1-year-old bamboos (2023), 2-year-old bamboos (2022) and 3-year-old bamboos (2021). Shrubs and weeds under the forest were removed by reclamation in May and June every year. In July, 450 kg/hm^2^ urea was applied in the furrow, and 20 cm was ploughed after fertilization to make it evenly mixed with the surface soil. The area is rich in understory vegetation communities. The shrub layer is mainly composed of *Rubus buergeri*, *Hydrangea davidi*, *Smilax china*, etc. The herb layer is mainly composed of *Elatostema involucratum*, *Impatiens balsamina*, *Pilea sinofasciat*, etc. The overall coverage of the forest is about 60%.

### 2.2 Experimental design

The field experiment plot is located in Shanshuping Village, Piaoba Village, Mugan Town, Daguan County, Zhaotong City. The elevation is 1–621 m, the slope is 22° east-south, and the slope is 21°. The soil is mountain yellow soil and medium soil layer. The experimental *Q. tumidinoda* forest was a plantation constructed by one-year-old container seedlings in November 2018. The average ground diameter of the bamboo forest was 1.05 cm, the average DBH was 0.56 cm, and the average height was 2.32 m. The density of untreated bamboo was 76222 plants/ha. In this study, a completely randomized block design was used to set up five treatments [(CK (0 kg/ha), K1 (75 kg/ha), K2 (150 kg/ha), K3 (225 kg/ha), K4 (300 kg/ha)], each treatment was repeated three times, a total of 15 experimental plots. The area of each plot is 3 m × 3 m, and the standard plot setting method is adopted: fixed stakes are set up in the four corners of the plot, and nylon rope is used to surround the plot, and the plot number is made at the same time. In order to eliminate the marginal effect and prevent the interference of bamboo rhizomes, all plots were set to avoid the forest edge, and a 2 m wide isolation belt was retained between adjacent plots. The potassium fertilizer was applied in mid-July 2023. The test potassium fertilizer was Sinochem Canada potassium chloride, and the K_2_O content was 60%. The potassium fertilizer was applied as base fertilizer at one time. The fertilization method was uniform hole application, the hole spacing was 1 m, the diameter of the hole was 20 cm, the depth was 20 cm, and the soil was covered after fertilization. The specific amount of fertilizer is shown in **Table 1**. The physical and chemical properties of soil before fertilization treatment are shown in **Table 2**.

**Table 1:** Potassium fertilizer treatments and application rates.

Treatment	Fertilizing amount of per unit area (kg/ha)	Area fertilization amount (g/9m^2^)
CK	0	0
K1	75	112.5
K2	150	225
K3	225	337.5
K4	300	450

**Table 2:** Soil physical and chemical properties before fertilization.

Soil horizon (cm)	pH	Organic matter (g/kg)	Total nitrogen (g/kg)	Total phosphorus (g/kg)	Total potassium (g/kg)	Hydrolysable nitrogen (mg/kg)	Available phosphorus (mg/kg)	Available potassium (mg/kg)
0-20	3.82	57.20	2.37	0.48	5.49	162.48	5.01	58.90
20-40	3.90	33.80	2.31	0.49	5.39	108.86	4.83	44.40

### 2.3 Sample collection and determination

#### 2.3.1 Sample collection

1. Bamboo shoots samples

From the end of March to the beginning of May 2024, *Q. tumidinoda* shoots were collected point-to-point in the experimental *Q. tumidinoda* forest (the height of bamboo shoots reached the local standard of 25–30 cm). Each sample was a point-mixed sample, and the fresh bamboo shoots sample weighed about 500 g. The non-edible bamboo shoots were removed immediately after the bamboo shoots were excavated and weighed. The height of bamboo shoots, the diameter of the lower end, the weight of single bamboo shoots with shell and the corresponding weight of single bamboo shoots without shell were measured one by one. After each collection of shoots, the number of shoots and the weight of fresh shoots in each experimental plot were counted and recorded accordingly. After the end of the bamboo shoot period, the data of each experimental plot were counted.

2. Soil samples

One year after the application of potassium fertilizer, soil samples were taken using the ‘ S ‘ type 5-point sampling method in each test plot. After removing the surface litter, 0~20 cm and 20~40 cm soil depths were taken. The collected soil samples were removed from stones, gravels, animal and plant residues and other impurities, and then the soil samples were fully mixed. The diagonal part of the sample was selected by the quartering method. The soil samples were placed in the indoor ventilation and dried in the shade. After drying, the soil samples were ground with a rolling pin, and then fully mixed. After passing 2 mm and 0.149 mm sieves, they were bagged and stored for use.

#### 2.3.2 Determination of indicators

1. Nutritional components of bamboo shoots

The bamboo shoot samples brought back to the laboratory were stripped of the bamboo shoot shell and the bamboo shoot coat. After rinsing with tap water and deionized water, the surface moisture was dried with a absorbent paper, and the inedible part was cut off. Then the bamboo shoots were cut into 1 cm×1 cm pieces, mixed and placed in an oven at 110 °C for 30 min, dried to constant weight at 70 °C, and then crushed by a high-speed grinder, passed through a 100-mesh sieve, and stored for later use. The basic nutrients measured mainly include: ash content was determined by total ash in food(GB 5009.4-2016); the content of crude fiber was determined by high temperature burning method(GB/T 5009.10-2003). Soluble sugar content was determined using a reduction iodometric method(NY/T 1278-2007), while starch content was analyzed through enzymatic hydrolysis(GB 5009.9-2016). Fat content was determined by Soxhlet extraction method(GB/T 5009.6-2016); the protein content was determined by Kjeldahl method(GB 5009.5-2010). Amino acid content was determined by protein hydrolysis method(GB 5009.124-2016), including essential amino acids and non-essential amino acids. All analyses were performed in three biological replicates.

2. Soil nutrient content

The soil pH value was determined by potentiometric method(LY/T 1239-1999). Soil organic matter was determined by potassium dichromate-external heating sulfuric acid oxidation method(LY/T 1237-1999). Soil total nitrogen was determined by Kjeldahl method(LY/T 1228-1999). Soil hydrolyzable nitrogen was determined by alkali hydrolysis-diffusion method(LY/T 1229-1999). Soil total phosphorus was determined by sodium hydroxide melting method(LY/T 1232-1999). Soil available phosphorus was determined by molybdenum antimony colorimetric method(LY/T 1233-1999). The total potassium in soil was determined by sodium hydroxide alkali fusion-flame photometer method(LY/T 1234-1999). Soil available potassium was extracted with ammonium acetate and determined by flame photometer(LY/T 1236-1999). All analyses were performed in three biological replicates.

### 2.4 Data processing and analysis

Microsoft Excel 2010 was used to process the survey data. IMB SPSS Statistics 25.0 was used to analyze the single factor (One-Way ANOVA) (α=0.05) variance analysis of bamboo shoot quality indicators and soil nutrients in *Q. tumidinoda* forest, and Origin 2022 software was used for mapping. At the same time, Duncan method was used for multiple comparisons of data with significant differences. The comprehensive evaluation of the nutritional quality of Qiongzhuea tumidinoda shoots was carried out by membership function method (Chen et al., 2009). The calculation methods of membership function and inverse membership function are as follows:

Membership function value calculation formula:

(1) U(Xi)=(Xi–Xmin)/(Xmax–Xmin)

Anti-membership function value calculation formula:

(2) $$U\left(X_{i}\right)=1–(X_{i}–X_{min})/(X_{max}–X_{min})$$

In the formula, *X_i_* is the actual measured value of each index; *X*_max_ and *X*_min_ were the maximum and minimum measured values of this index.

## 3 Results

### 3.1 Effects of potassium application on proximate composition of *Q. tumidinoda* shoots

#### 3.1.1 Ash and crude fiber content

**Figure 1:**
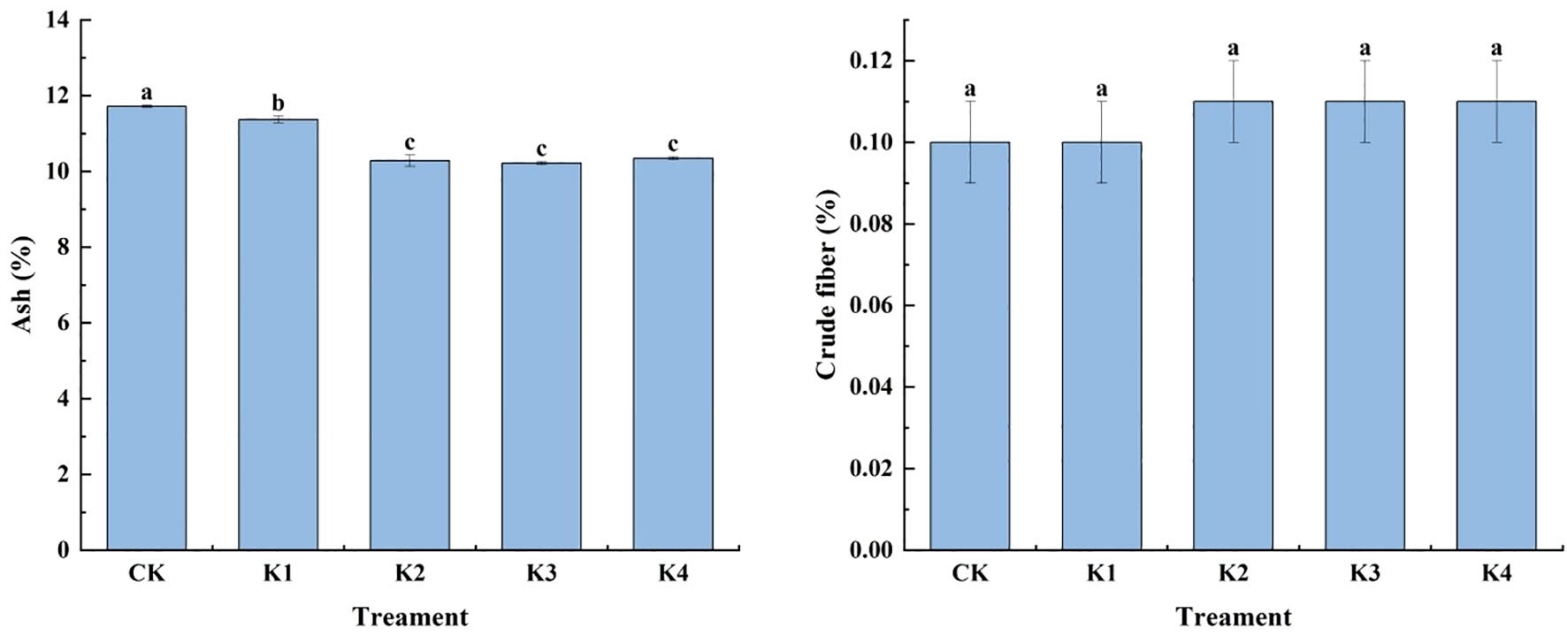
Effects of different potassium applications on ash and crude fiber content of *Q. tumidinoda* shoots. Different lowercase letters indicate significant differences between different treatments. (N = 3, *P*<0.05).

The application of potassium fertilizer had no significant effect on the crude fiber content of *Q. tumidinoda* shoots. Except for K1 treatment, the crude fiber content of *Q. tumidinoda* shoots in the other three treatments was higher than that of CK (**Figure 1**). The crude fiber content of *Q. tumidinoda* a shoots in CK and K1 treatments was 0.1 g per 100 g, while the crude fiber content of *Q. tumidinoda* shoots in K2, K3 and K4 treatments was 0.11 g. The application of potassium fertilizer had a certain effect on the ash content of *Q. tumidinoda* shoots. With the increase of potassium fertilizer application, the ash content of *Q. tumidinoda* shoots decreased significantly (*P*<0.05), but it was significantly lower than that of CK and K1 treatments (*P*<0.05) (**Figure 1**).

#### 3.1.2 Protein and crude fat content

**Figure 2:**
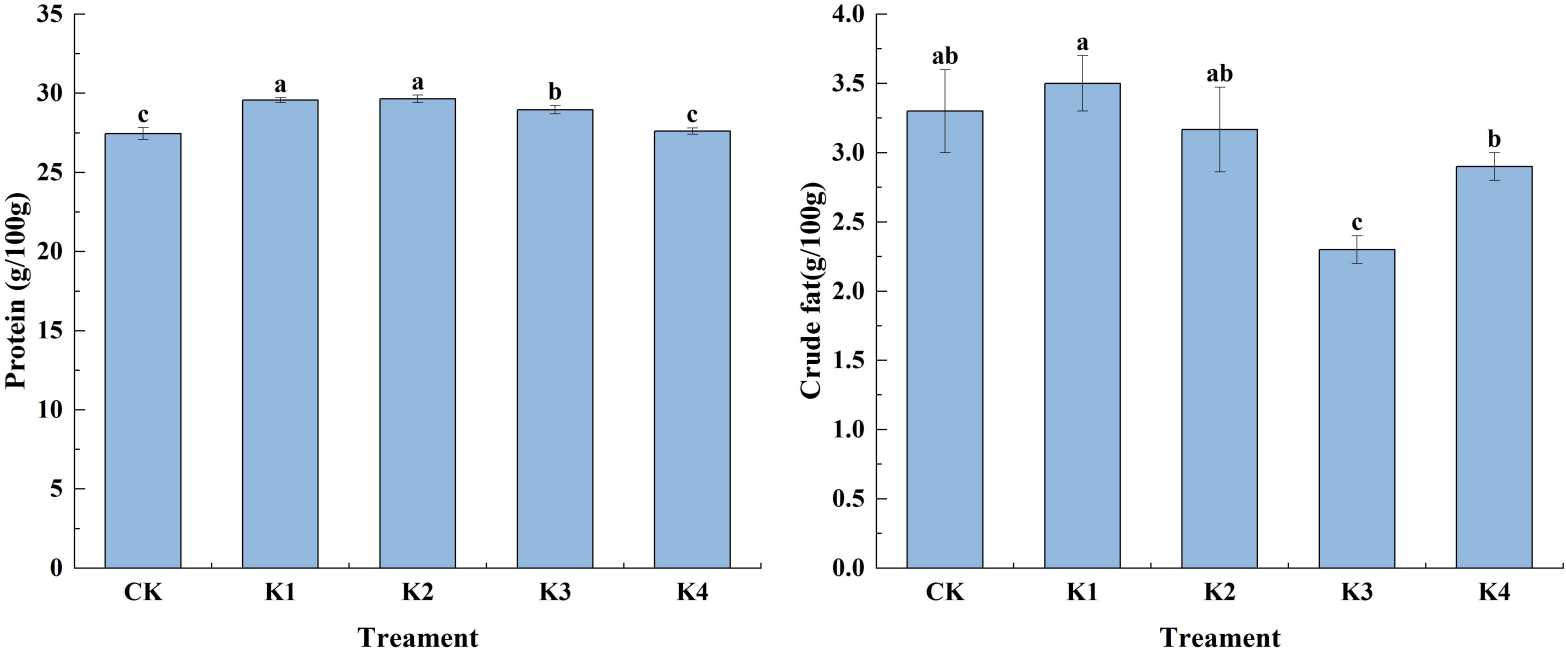
Effects of different potassium applications on carbohydrate and crude fat content of *Q. tumidinoda* shoots. Different lowercase letters indicate significant differences between different treatments. (N = 3, *P*<0.05).

The application of potassium fertilizer significantly increased the protein content of *Q. tumidinoda* shoots. With the increase of potassium application, the protein content of *Q. tumidinoda* shoots increased first and then decreased (**Figure 2**). The protein content of *Q. tumidinoda* shoots treated with K2 was the highest, and the protein content of *Q. tumidinoda* shoots treated with K1, K2 and K3 was significantly higher than that of CK (*P*<0.05). Compared with CK, K1, K2, K3 and K4 treatments increased by 7.72%, 8.03%, 5.54% and 0.61%, fragment. The application of potassium fertilizer (except K1 treatment) significantly reduced the crude fat content of *Q. tumidinoda* shoots (*P*<0.05). With the increase of potassium application rate, the crude fat content of *Q. tumidinoda* shoots decreased first and then increased, and the crude fat content of *Q. tumidinoda* shoots treated with K3 was the lowest (**Figure 2**). The crude fat content of *Q. tumidinoda* shoots in each treatment was K1 > CK > K2 > K4 > K3. The crude fat content of *Q.* tumidinoda shoots treated with K3 decreased by 43.48%, 52.17%, 39.13% and 26.09% respectively compared with CK, K1, K2 and K4 treatments.

#### 3.1.3 Soluble sugar and starch content

**Figure 3:**
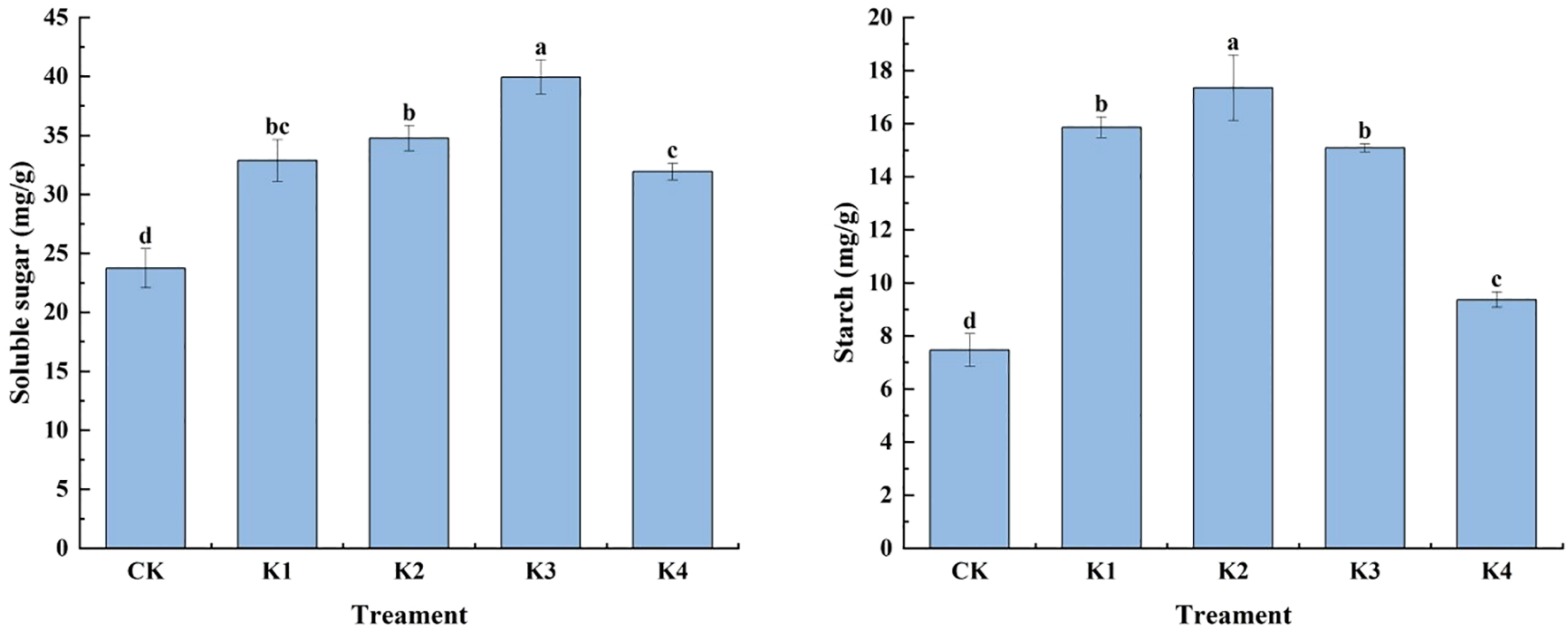
Effects of different potassium applications on the soluble sugar and starch content of *Q. tumidinoda* shoots. Different lowercase letters indicate significant differences between different treatments. (N = 3, *P*<0.05).

As shown in **Figure 3**, the application of potassium fertilizer had a significant effect on the soluble sugar and starch content of *Q. tumidinoda* (*P*<0.05). With the increase of potassium fertilizer application rate, the soluble sugar content of bamboo shoots increased first and then decreased. The soluble sugar content of K3 treatment was the highest, which was 39.94 mg/g, followed by K2 treatment, which was 34.76 mg/g. The soluble sugar contents of K1, K4 and CK treatments were 32.87 mg/g, 31.92 mg/g and 23.73 mg/g, respectively. Starch and soluble sugar had the same change rule. With the increase of potassium fertilizer application rate, the starch content of *Q. tumidinoda* shoots increased first and then decreased. The starch content of each potassium treatment was 1.88~9.88 mg/g higher than that of CK. Under K2 treatment, the starch content of bamboo shoots was the highest, and K2 treatment was 132.26%, 9.39%, 14.99% and 85.36% higher than CK, K1, K3 and K4 treatments, respectively. The results showed that the appropriate amount of potassium fertilizer could increase the soluble sugar and starch content of *Q. tumidinoda* shoots, but with the increase of potassium fertilizer application, the soluble sugar and starch content no longer increased, but showed a downward trend.

#### 3.1.4 Changes of amino acid content in *Q. tumidinoda* shoots

It can be seen from **Table 3** that the application of potassium fertilizer had no significant effect on the content of isoleucine and essential amino acids (*P*>0.05). The content of isoleucine in CK was the highest and that in K2 was the lowest. The total content of essential amino acids increased first and then decreased. Except for K4 treatment, the content of essential amino acids in other treatments was higher than that of CK. There were significant effects on other types of amino acids and the proportion of total amino acids and essential amino acids (*P*<0.05). For the percentage of essential amino acids, the general pattern was that the percentage of essential amino acids was lower than that of CK for all fertilized treatments, in descending order of CK>K1>K3>K2>K4; there was a significant difference between CK and all the other treatments (*P*<0.05), whereas the differences were not significant (*P*>0.05) among K1, K2, K3 and K4 treatments.

**Table 3:** Amino acid species and content of bamboo shoots (g/100g).

Kinds of amino acid	Treatment				
	CK	K1	K2	K3	K4
Threonine ^*^	1.06 ± 0.02c	1.08 ± 0.01bc	1.23 ± 0.01a	1.11 ± 0.03b	1.07 ± 0.04c
Valine ^*^	1.40 ± 0.01c	1.47 ± 0.03ab	1.41 ± 0.03c	1.48 ± 0.01a	1.45 ± 0.01b
Methionine ^*^	0.27 ± 0.01b	0.30 ± 0.01a	0.28 ± 0.01b	0.28 ± 0.01b	0.28 ± 0.01b
Isoleucine ^*^	1.22 ± 0.08a	1.20 ± 0.02a	1.15 ± 0.01a	1.21 ± 0.01a	1.17 ± 0.01a
Leucine ^*^	1.81 ± 0.02c	1.90 ± 0.03a	1.91 ± 0.02a	1.88 ± 0.01ab	1.86 ± 0.02b
Phenylalanine ^*^	1.12 ± 0.01c	1.19 ± 0.01a	1.20 ± 0.01a	1.17 ± 0.01b	1.17 ± 0.01b
Lysine ^*^	1.53 ± 0.02e	1.68 ± 0.02a	1.65 ± 0.03b	1.62 ± 0.01c	1.57 ± 0.01d
Aspartic acid	3.27 ± 0.02c	3.21 ± 0.04a	3.44 ± 0.05a	3.56 ± 0.01a	3.53 ± 0.02a
Serine	0.90 ± 0.04d	0.97 ± 0.01b	1.22 ± 0.01a	0.89 ± 0.02d	0.93 ± 0.01c
Glutamic acid	2.87 ± 0.01d	3.02 ± 0.05ab	2.99 ± 0.04b	3.05 ± 0.01a	2.94 ± 0.01c
Glycine	1.40 ± 0.01c	1.46 ± 0.03a	1.37 ± 0.02d	1.45 ± 0.02ab	1.43 ± 0.01b
Alanine	1.99 ± 0.01e	2.14 ± 0.04b	2.22 ± 0.03a	2.08 ± 0.01c	2.04 ± 0.02d
Tyrosine	0.77 ± 0.01c	0.87 ± 0.01a	0.88 ± 0.01a	0.81 ± 0.01b	0.88 ± 0.04a
Histidine	0.49 ± 0.01c	0.55 ± 0.01b	0.64 ± 0.01a	0.53 ± 0.01b	0.55 ± 0.05b
Arginine	1.33 ± 0.01d	1.42 ± 0.02ab	1.44 ± 0.02a	1.41 ± 0.01b	1.38 ± 0.01c
Proline	1.15 ± 0.01c	1.19 ± 0.02a	1.20 ± 0.01a	1.18 ± 0.01ab	1.17 ± 0.01b
Essential amino acid	8.69 ± 0.29a	8.82 ± 0.08a	8.82 ± 0.07a	8.76 ± 0.01a	8.56 ± 0.01a
Total amino acids	22.50 ± 0.10d	23.67 ± 0.15b	24.23 ± 0.21a	23.73 ± 0.06b	23.37 ± 0.15c
The proportion of essential amino acids	38.63 ± 1.43a	37.25 ± 0.12b	36.40 ± 0.08b	36.91 ± 0.13b	36.65 ± 0.22b

Different lowercase letters indicate that the same amino acid content in different treatments is significantly different (*P*<0.05). ‘ * ‘ denotes essential amino acids.

It can be seen from **Table 4** that the amount of potassium fertilizer had no significant effect on the bitter amino acid content of *Q. tumidinoda*(*P*>0.05), and had significant effects on other indicators (*P*<0.05). The order of umami amino acid content was: K3>K4>K2>K1>CK; with the increase of potassium application rate, the content of sweet amino acids and aromatic amino acids increased first and then decreased. The content of sweet amino acids under K1, K2, K3 and K4 treatments increased by 5.22%, 11.21%, 3.23% and 2.15% respectively compared with CK, and the content of aromatic amino acids increased by 8.99%, 10.05%, 5.29% and 8.47% respectively compared with CK. The bitter amino acid content was the lowest under K4 treatment and the highest under K1 treatment. The proportion of sweet amino acids increased first and then decreased. The proportion of umami amino acids increased compared with CK treatment. The proportion of bitter amino acids was K3>K4>K1>CK>K2. The proportion of aromatic amino acids increased compared with CK (except K3). The maximum proportion of aromatic amino acids appeared in K1 treatment, and the minimum value appeared in K3 treatment.

**Table 4:** Flavor-presenting amino acids and their ratios in bamboo shoots.

Item	Treatment				
	CK	K1	K2	K3	K4
Umami amino acid	6.15 ± 0.02b	6.23 ± 0.07c	6.43 ± 0.06b	6.61 ± 0.02a	6.47 ± 0.01b
Sweet amino acids	6.51 ± 0.05d	6.85 ± 0.06c	7.24 ± 0.05a	6.72 ± 0.05c	6.65 ± 0.01c
Bitter amino acid	6.59 ± 0.31a	6.62 ± 0.05a	6.54 ± 0.06a	6.56 ± 0.03a	6.52 ± 0.01a
Aromatic amino acids	1.89 ± 0.01d	2.06 ± 0.01ab	2.08 ± 0.02a	1.99 ± 0.01c	2.05 ± 0.02b
Umami amino acids ratio	27.03 ± 1.43b	27.24 ± 0.16b	29.36 ± 0.08a	28.56 ± 0.16ab	28.24 ± 0.14ab
Sweet amino acids ratio	28.92 ± 0.10b	28.94 ± 0.09b	29.86 ± 0.09a	28.33 ± 0.15b	28.47 ± 0.18c
Bitter amino acid ratio	22.78 ± 1.13ab	22.88 ± 0.15ab	21.90 ± 0.25b	23.15 ± 0.19a	22.91 ± 0.15ab
Aromatic amino acids ratio	7.01 ± 0.35ab	7.55 ± 0.06a	7.07 ± 0.07ab	6.96 ± 0.07b	7.25 ± 0.03ab

Different lowercase letters indicate that the same amino acid content or proportion of different treatments is significantly different (P<0.05).

### 3.2 Effects of potassium fertilizer application on soil nutrient content of *Q. tumidinoda* forest

#### 3.2.1 Changes of soil pH and organic matter content in different treatments

**Figure 4:**
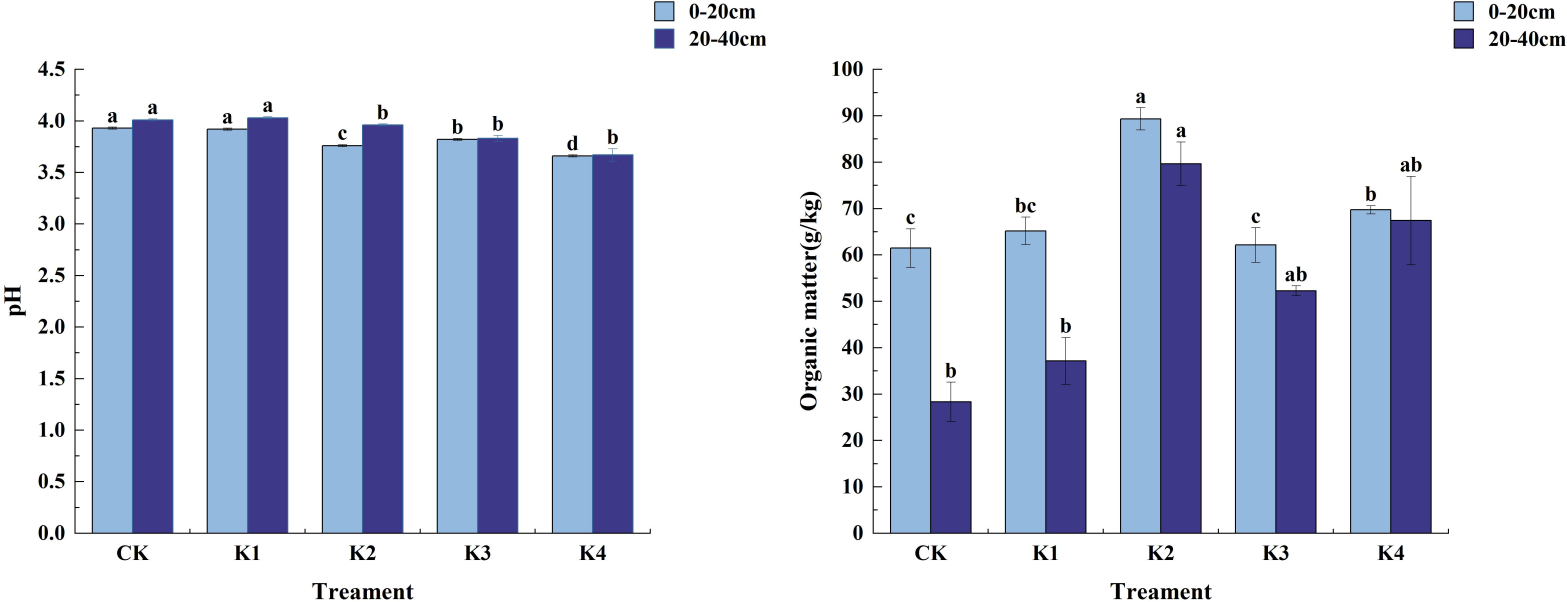
Soil pH and organic matter content under different potassium application treatments. Different lowercase letters indicate significant differences between different treatments in the same soil layer. (N = 3, *P*<0.05).

As shown in **Figure 4**, the soil pH value of different potassium treatments was between 3.66 and 4.03, and the soil organic matter content increased. In the 0~20 cm soil layer, the soil pH value decreased after the application of potassium fertilizer. The soil pH value in each treatment was CK>K1>K3>K2>K4, and the fertilization treatments decreased by 0.25%, 2.80%, 4.33% and 6.87% respectively compared with CK. The soil organic matter content was the highest under K2 treatment, which was significantly different from CK, K1, K3 and K4 treatments (*P*<0.05). There was no significant difference between K1 and K4 treatments (*P*>0.05), and there was no significant difference between CK and K1 and K3 treatments (*P*>0.05). In the 20~40 cm soil layer, the soil pH value showed a trend of increasing first and then decreasing. The maximum soil pH value of K1 treatment was 4.03, and the minimum soil pH value of K4 treatment was 3.67. In the 20~40 cm soil layer, The soil organic matter content of K2 treatment was the highest, which was 79.66 g/kg, followed by K4 treatment, which was 67.41 g/kg. The soil organic matter content of K3, K1 and CK treatment was 52.26 g/kg, 37.14 g/kg and 28.32 g/kg, respectively. The K2 treatment was 114.49%, 52.44%, and 18.17% higher than the K1, K3, and K4 treatments, respectively.

#### 3.2.2 Changes of soil total nitrogen and hydrolysable nitrogen content under different treatments

**Figure 5:**
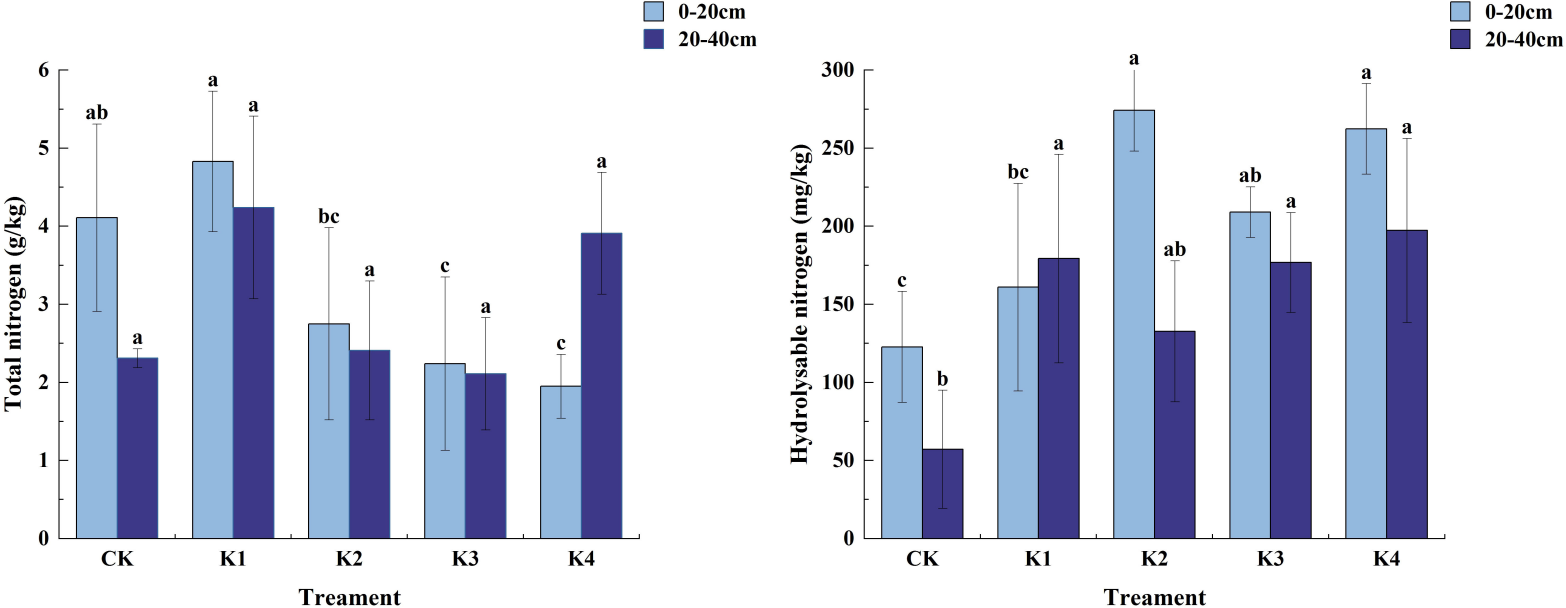
Nitrogen content in soil treated with different potassium treatments. Different lowercase letters indicate significant differences between different treatments in the same soil layer. (N = 3, *P*<0.05).

It can be seen from **Figure 5** that the total nitrogen content of soil in different fertilization treatments decreased with the increase of soil depth (except for K4 treatment), but the content of soil hydrolyzed nitrogen increased significantly. Except that the content of hydrolyzed nitrogen in K1 treatment was higher than that in 0~20 cm soil layer, the content of hydrolyzed nitrogen in other fertilization treatments was lower than that in 0~20 cm soil layer.

In the 0~20 cm soil layer, the application of potassium fertilizer had a significant effect on soil total nitrogen content (*P*<0.05). With the increase of potassium fertilizer application, soil total nitrogen content increased first and then decreased. Among them, the total nitrogen content of K1 treatment was the highest, which was 4.83 g/kg, and that of K4 treatment was the lowest, which was 1.95 g/kg. Except that there was no significant difference between K1 and CK (*P*>0.05), there were significant differences between the other three treatments and CK (*P*<0.05). The maximum content of hydrolyzable nitrogen appeared in K2 treatment, which was 274.28 mg/kg, followed by K4 treatment, which was 262.30 mg/kg. K3 and K1 were 208.99 mg/kg and 160.95 mg/kg, respectively, which were 31.11%, 123.43%, 70.25% and 113.67% higher than CK, respectively. In the 20~40 cm soil layer, there was no significant difference in total nitrogen content between treatments (P>0.05), and the content was K1>K4>K2>CK>K3. The differences between the fertilizer treatments (except K2 treatment) and CK were significant (*P*<0.05) and the differences in hydrolyzed nitrogen content between the fertilizer treatments were not significant (*P*>0.05). The content of hydrolyzed nitrogen in each fertilization treatment was K4>K1>K3>K2>CK, which increased by 213.65%, 132.23%, 209.31% and 245.17% respectively compared with CK.

#### 3.2.3 Changes of soil total phosphorus and available phosphorus content under different treatments

**Figure 6:**
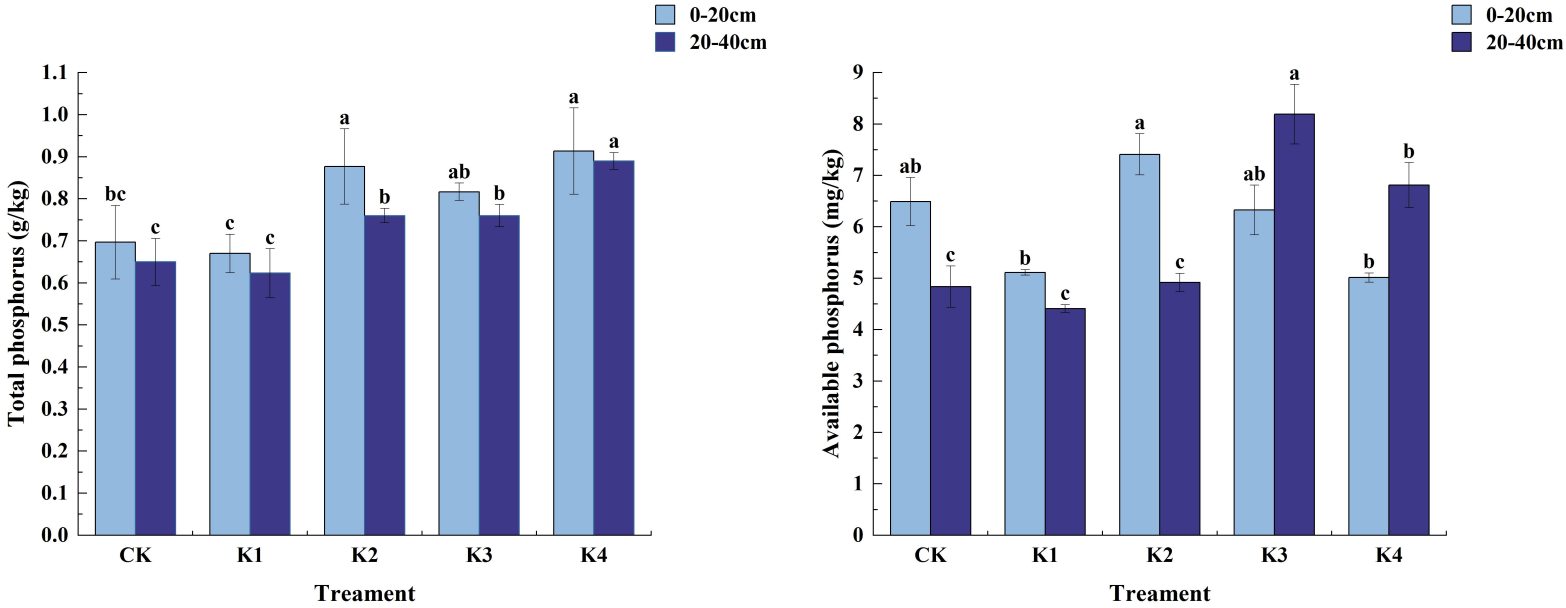
Phosphorus content in soil treated with different potassium treatments. Different lowercase letters indicate significant differences between different treatments in the same soil layer. (N = 3, *P*<0.05).

It can be seen from **Figure 6** that with the increase of potassium fertilizer application rate, the total phosphorus content of soil decreased first and then increased. In different fertilization treatments, the total phosphorus content in 0~20 cm soil layer was the highest, and the total phosphorus content in K4 treatment was the highest in both soil layers, reaching 0.91 g/kg in 0~20 cm soil layer and 0.89 g/kg in 20~40 cm soil layer.

In the 0 ~ 20 cm soil layer, the total phosphorus content of the soil treated with K1 decreased, and the total phosphorus content of the soil treated with other fertilization treatments increased. The total phosphorus content of the soil between the treatments was K4 > K2 > K3 > CK > K1. The application of potassium fertilizer had a significant effect on soil available phosphorus content (*P*<0.05). K2 treatment was significantly higher than K1 and K4 treatments (*P*<0.05), and had no significant difference with CK and K3 treatments (*P*>0.05).The content of available phosphorus in soil was the highest in K2 treatment, which was 7.41 mg/kg, and the content of available phosphorus in K4 treatment was the lowest, which was 5.01 mg/kg. Compared with CK, K3, K1 and K4 decreased by 2.52%, 21.21% and 22.75% fragment. In the 20~40 cm soil layer, the difference between K4 treatment and the other four treatments was significant (*P*<0.05). The application of potassium fertilizer had a certain effect on the content of available phosphorus. There was a significant difference between K3 and K4 (*P*<0.05), and it was significantly different from CK, K1 and K2 (*P*<0.05). However, there was no significant difference between CK, K1 and K2 (*P*>0.05). The content of available phosphorus in each treatment was K3>K4>K2>CK>K1.

#### 3.2.4 Changes of soil total potassium and available potassium content under different treatments

**Figure 7:**
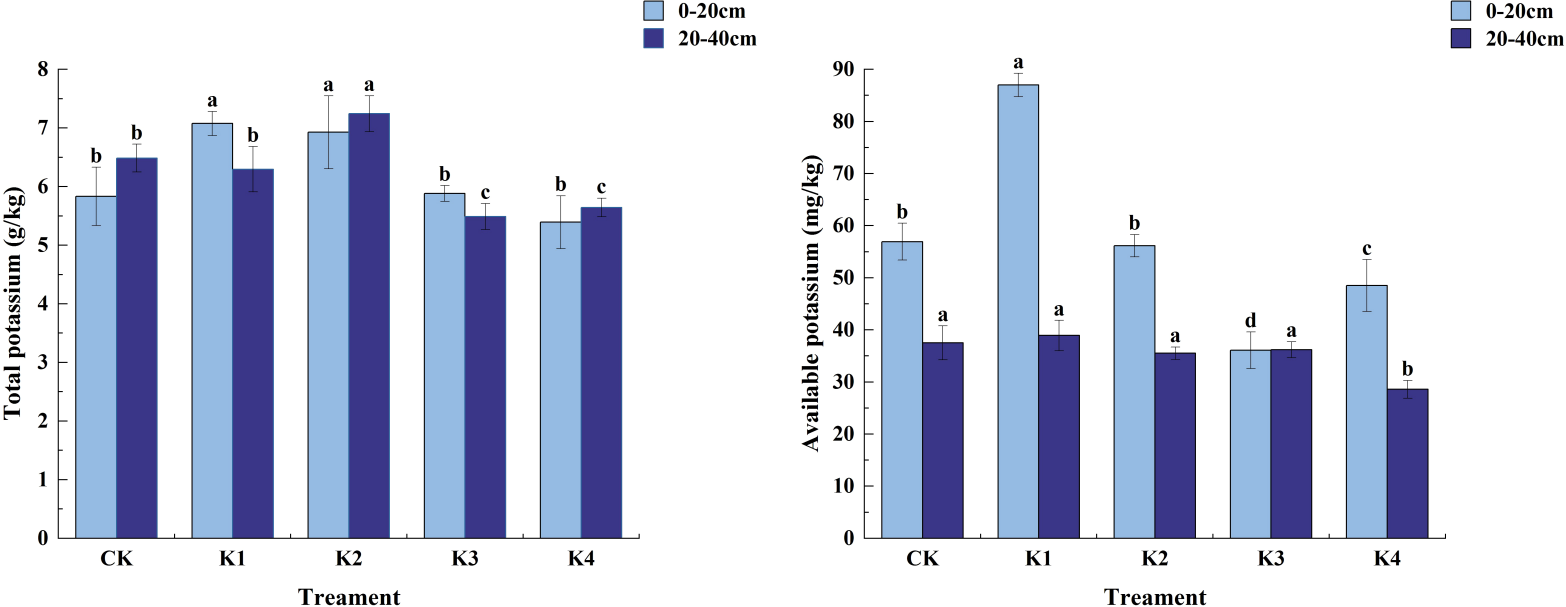
Potassium content in soil treated with different potassium application. Different lowercase letters indicate significant differences between different treatments in the same soil layer. (N = 3, *P*<0.05).

**Figure 7** shows that with the increase of potassium fertilizer application rate, the total potassium content of soil increased first and then decreased. In the 0~20 cm soil layer, the soil total potassium content of K1 and K2 treatments was significantly higher than that of CK treatment (*P*<0.05), and the difference between K3 and K4 treatments and CK treatment was not significant (*P*>0.05). Among them, the total potassium content of K1 treatment was the highest, 7.08 g/kg, and that of K4 treatment was the lowest, 5.39 g/kg. Except for K4 treatment, K1, K2 and K3 treatments increased by 21.31%, 18.74% and 0.86%, respectively, compared with CK treatment. There was a significant effect on soil available potassium content (*P*<0.05). Except that there was no significant difference between K2 and CK (*P*>0.05), the other three treatments were significantly different from CK (*P*<0.05). The maximum available potassium content appeared in K1 treatment, which was 87.02 mg/kg. K2, K3 and K4 were 56.16 mg/kg, 36.09 mg/kg and 48.51 mg/kg, respectively, which were 1.36%, 36.59% and 14.81% lower than CK, respectively. Significant differences (*P*<0.05) in whole potassium content among treatments were observed in the 20~40 cm soil layer, with the magnitude of the content being K2>CK>K1>K4>K3. For quick-acting potassium content only significant differences (*P*<0.05) were found between K4 and CK treatments, while none of them were significant (*P*>0.05) between K1, K2, K3 and CK. The magnitude of quick-acting potassium content among fertilization treatments was K1>CK>K3>K2>K4 in the order of K1>CK>K3>K2>K4, K1 increased by 3.73% compared with CK, and K3, K2, and K4 decreased by 5.42%, 3.55%, and 23.80% compared with CK, respectively.

### 3.3 Correlation analysis between soil pH, nutrient index and bamboo shoot quality index

**Figure 8:**
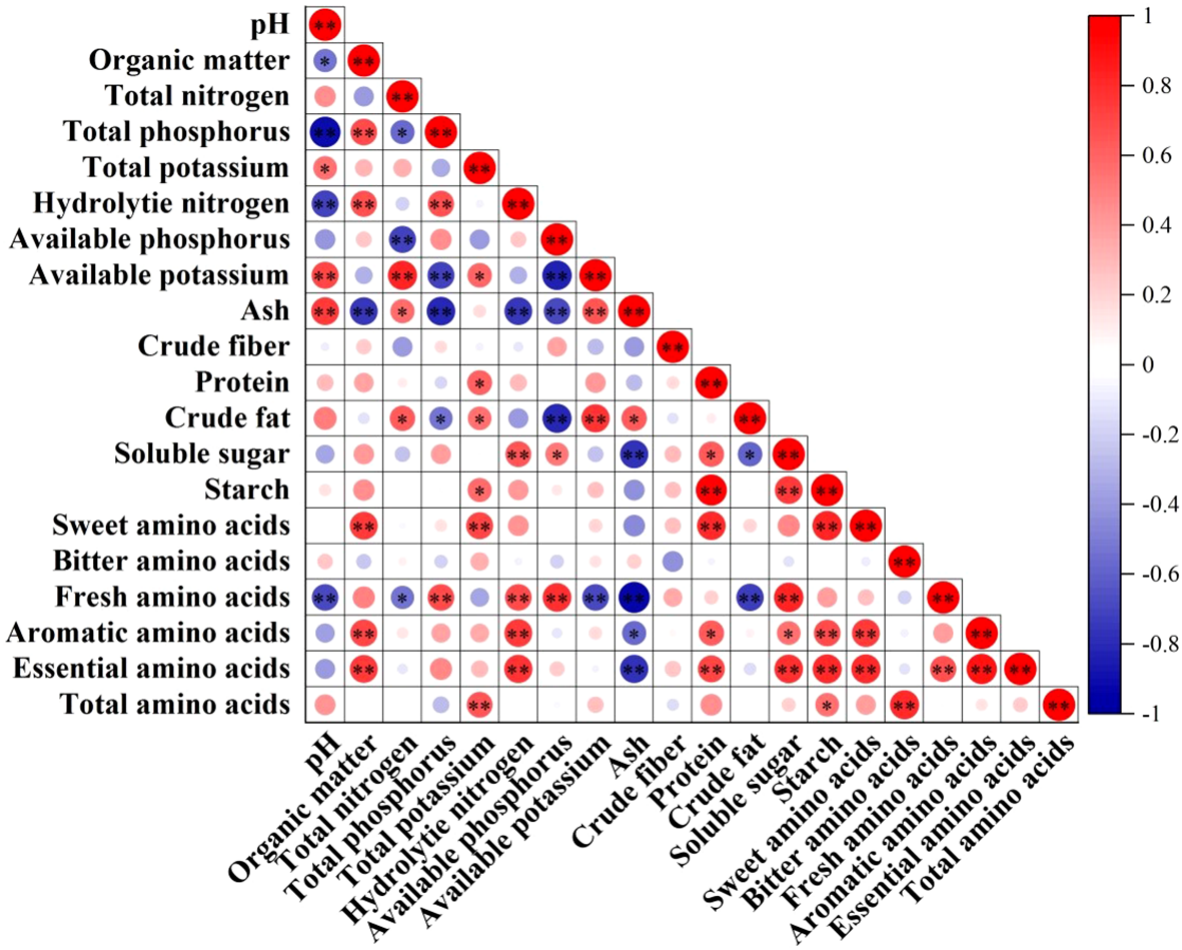
The correlation analysis results of soil pH and nutrient indexes with bamboo shoot quality indexes.’*’on error bars indicate significant differences at *P*<0.05,’**’on error bars indicate significant differences at *P*<0.01.

The correlation analysis between soil pH and nutrient index and bamboo shoot quality index is shown in **Figure 8**. Soil pH was significantly positively correlated with ash content of bamboo shoots, and significantly negatively correlated with aromatic amino acid content. Soil organic matter content was significantly negatively correlated with ash content, and significantly positively correlated with sweet amino acid content, aromatic amino acid content and total amino acid content. Soil total nitrogen content was significantly positively correlated with ash content and crude fat content, and significantly negatively correlated with aromatic amino acids. Soil total phosphorus content was significantly negatively correlated with ash content, significantly negatively correlated with crude fat content, and significantly positively correlated with aromatic amino acid content. Soil total potassium content was significantly positively correlated with protein content, crude fat content and starch content, and was significantly positively correlated with sweet amino acid content and essential amino acid content. Soil hydrolyzable nitrogen content was significantly negatively correlated with ash content, and significantly positively correlated with soluble sugar content, umami amino acid content, aromatic amino acid content and total amino acid content. Soil available phosphorus content was significantly negatively correlated with ash content and crude fat content, significantly positively correlated with soluble sugar content, and significantly positively correlated with aromatic amino acid content. Soil available potassium content was significantly positively correlated with ash content and crude fat content, and significantly negatively correlated with aromatic amino acid content.

### 3.4 Principal component analysis of quality indexes of *Q. tumidinoda* shoots

When performing principal component analysis, the principal components were screened according to the criteria of eigenvalue > 1 and cumulative variance contribution rate > 85% (Bian et al., 2024). It can be seen from **Table 5** that three principal components were extracted in this study, and their eigenvalues were 5.927, 2.806 and 1.692, respectively. The corresponding variance contribution rates were 49.388%, 23.384% and 14.098%, respectively, and the cumulative contribution rate was 86.870%, which met the requirements of statistical analysis. The indexes with higher load in the first principal component were protein, soluble sugar, starch, total amino acid, umami amino acid, bitter amino acid and aromatic amino acid. In the second principal component, protein, crude fat, essential amino acids and sweet amino acids had higher loads. In the third principal component, the higher load is essential amino acids and sweet amino acids.

**Table 5:** Principal component analysis of bamboo shoot quality indicators.

Norm	Load coefficient		
	PC1	PC2	PC3
Ash	-0.817	0.471	-0.119
Crude fiber	0.481	-0.585	-0.126
Protein	0.750	0.510	-0.165
Crude fat	-0.338	0.648	-0.599
Soluble sugar	0.897	-0.197	0.206
Starch	0.864	0.425	-0.012
Essential amino-acid	0.275	0.699	0.621
Total amino acids	0.959	0.086	-0.121
Fresh Amino Acids	0.779	0.444	-0.274
Sweet Amino Acids	-0.137	0.518	0.787
Bitter amino acids	0.756	-0.567	0.251
Aromatic amino acids	0.781	0.246	-0.274
Eigenvalue	5.927	2.806	1.692
Variance contribution rate (%)	49.388	23.384	14.098
Cumulative proportion in anova (%)	49.388	72.772	86.870

### 3.5 Comprehensive evaluation of potassium fertilizer on the quality of *Q. tumidinoda* shoots

In the correlation analysis, if an index is significantly correlated with two or more indexes (the absolute value of the correlation coefficient exceeds 0.5), it shows that there is a strong correlation between the index and the quality of bamboo shoots, and it can be determined that it has a greater impact on the quality. Based on principal component analysis, correlation of each index and other influencing factors, ash, crude fiber, protein, crude fat, soluble sugar, starch, total amino acid, sweet amino acid and aromatic amino acid were finally selected to calculate the membership function value (Jing et al., 2020). According to Equations 1, 2, the average membership function values of Qiongzhuea tumidinoda shoots under five potassium treatments were calculated, and the quality was ranked (**Table 6**). The comprehensive ranking is as follows : K2 > K1 > K3 > K4 > CK.

**Table 6:** Comprehensive evaluation scores of *Q. tumidinoda* shoots treated with different potassium application levels.

Treatment	Mean membership index value	Sort
CK	0.23	5
K1	0.53	2
K2	0.57	1
K3	0.39	3
K4	0.32	4

## 4 Discussion

### 4.1 Effects of potassium fertilizer application on nutritional quality of *Q. tumidinoda* shoots

The quality evaluation indexes of bamboo shoots include nutrient content and flavor substance content. The nutrients required for the growth of bamboo shoots come from the photosynthesis of mother bamboo and the nutrients absorbed by roots from the soil. The growth and material accumulation of bamboo shoots can be achieved by fertilization to provide sufficient nutrients for the growth of bamboo shoots (Ke et al., 2023). The main component of ash is inorganic salt, and inorganic salt is one of the six major nutrients. Therefore, ash content can be used as one of the reference indexes for evaluating food nutrition. Ash content can reflect the characteristics of selective absorption and accumulation of mineral nutrients by plants (Li et al., 2018). The determination of ash content can understand the nutritional content of bamboo shoots to a certain extent, and has a better reference role in the nutritional value and use value of bamboo shoots (Dong and Li, 2021). The results of this study showed that the ash content of *Q. tumidinoda* shoots decreased significantly after the application of potassium fertilizer, indicating that the application of potassium fertilizer had a negative correlation with the accumulation of ash content in *Q. tumidinoda* shoots. The reason may be that the application of potassium fertilizer reduced the accumulation ability of *Q. tumidinoda* to calcium, magnesium and other elements, resulting in a decrease in total mineral content (Wang, 2021). Crude fiber includes cellulose, hemicellulose and lignin, and can not be digested and absorbed by the human body (Li et al., 2018). However, it is of great significance to the human body, which can promote digestion and regulate intestinal function (Yang et al., 2021). In this study, the crude fiber content of *Q. tumidinoda* shoots was not significantly different among different treatments, indicating that the application of potassium fertilizer had little effect on the crude fiber content of *Q. tumidinoda* shoots, which was inconsistent with the results of Rong Jundong et al (Rong et al., 2017). study on the effects of fertilization on the nutrients and nutrients of *Pleioblastus amarus* shoots.

Soluble sugars and starch together constitute non-structural carbohydrates (NSC), and plant vegetative tissues store nutrients in the form of NSC, which is extremely important for plant growth and development (Zhang, 2021). Soluble sugar content affects the taste and nutritional value of bamboo shoots. In this study, the application of potassium fertilizer can significantly increase the content of soluble sugar, and the change trend is first increased and then decreased. The most suitable amount of potassium is under K3 treatment. Potassium enhances the utilization of nitrogen, strengthens photosynthesis in bamboo, accumulates photosynthetic products, and increases the soluble sugar content (Nong et al., 2025). Crude fat is an important part of the human body and one of the main energy-supplying substances (Niu et al., 2017). Bamboo shoot is a kind of food with low fat content. The fat content of various bamboo shoots is generally 0.30% -3.97% (Ke et al., 2023). In this study, the crude fat content of *Q. tumidinoda* shoots was 2.2% -3.7%. The difference between CK and K1 and K2 treatments was not significant, but the difference between CK and K3 and K4 treatments was significant, indicating that low potassium treatment had little effect on the fat accumulation of *Q. tumidinoda* shoots. The synthesis of crude fat is a highly energy-consuming process, which requires the participation of ATP. In this study, the ash content of bamboo shoots decreased after the application of potassium fertilizer. It may be that the application of potassium inhibited the absorption of calcium and magnesium ions by *Q. tumidinoda*, and magnesium ions affected the synthesis and utilization of ATP, so there was not enough energy to provide for the synthesis of crude fat.

As an important component of all cells and tissues in the human body, proteins are involved in most biological processes (Li et al., 2023). Studies have shown that fertilization can significantly promote the accumulation of protein content in bamboo shoots (Zheng et al., 2004). The results of this study showed that the application of potassium fertilizer could significantly increase the protein content of *Q. tumidinoda* shoots, and the change rule was that the protein content of *Q. tumidinoda* shoots increased first and then decreased with the increase of potassium fertilizer application. Another important criterion for evaluating the quality of bamboo shoots is amino acids, including the type and content of amino acids (Li et al., 2021). Studies have shown that the application of fertilizers significantly promoted the accumulation of umami amino acids in bamboo shoots, and also increased the content of umami and sweet amino acids (Ke et al., 2023). The results of this study are consistent with the results of previous studies. In this study, with the application of potassium fertilizer, the proportion of umami amino acids in *Q. tumidinoda* shoots increased overall. For sweet amino acids, with the increase of potassium fertilizer application, the proportion of sweet amino acids increased first and then decreased, indicating that excessive potassium fertilizer in turn affected the accumulation of sweet amino acids, while the proportion of bitter and aromatic amino acids decreased overall, indicating that the amino acid composition of *Q. tumidinoda* shoots changed after potassium fertilizer application, and *Q. tumidinoda* shoots were more umami and sweet.

### 4.2 Effect of potassium fertilizer application on soil nutrient content

Soil is the basis of seedling growth, and its nutrient status directly affects the growth and development of plants. The application of potassium fertilizer can significantly increase the soil nutrient content and optimize the nutrient absorption and utilization efficiency of seedlings. Among them, soil organic matter and available nitrogen, phosphorus, and potassium are the main indicators for evaluating soil fertility. Their content not only reflects the soil’s nutrient storage and supply capacity, but also is a key determinant of plant growth potential. Yang (Yang, 2013) studied the effect of potassium fertilizer on the vegetative growth and fruiting of *Camellia oleifera*. It was found that the application of higher potassium in autumn was helpful to the accumulation of soil total nitrogen, organic matter and available potassium. The results of this study showed that except for total nitrogen content, the application of potassium fertilizer increased the contents of soil organic matter, hydrolyzed nitrogen, total phosphorus, available phosphorus, total potassium and available potassium to a certain extent (compared with CK), but the increase of each nutrient index was different. It shows that the application of potassium fertilizer is indeed beneficial to improve soil fertility and improve soil nutrient content, and the application amount of potassium fertilizer is closely related to soil nutrient content. Therefore, reasonable regulation of potassium fertilizer input is of great significance to soil nutrient management.

Studies have shown that with the increase of potassium application rate, the content of soil hydrolyzed nitrogen decreased, the content of total nitrogen remained stable, and the content of total potassium, available potassium and organic matter continued to increase (Wang et al., 2020). The results of this study were consistent with the existing research conclusions, indicating that there was a significant dose-effect relationship between potassium fertilizer application rate and soil nutrient accumulation and nutrient absorption efficiency of *Q. tumidinoda* clones. When the amount of potassium fertilizer deviates from the appropriate range (whether too high or too low), it is not conducive to soil nutrient accumulation, which in turn leads to a decrease in nutrient absorption efficiency of plants. This study showed that the content of soil hydrolyzed nitrogen was higher than that of the control (CK) under different potassium fertilizer application rates, indicating that increasing potassium fertilizer under potassium deficiency conditions can promote the absorption and utilization of soil nitrogen by crops. However, the content of soil hydrolysable nitrogen in the K3 treatment group showed a downward trend, which indicated that excessive application of potassium fertilizer may inhibit the absorption efficiency of nitrogen by crops.

The results showed that the application of potassium fertilizer on the basis of nitrogen and phosphorus fertilizer could significantly increase the content of organic matter, available and total nitrogen, phosphorus and potassium in soil, especially the content of available potassium in soil (Zhang et al., 2012). The results of this study showed that with the increase of potassium fertilizer application rate, the organic matter content in the soil of *Q. tumidinoda* forest increased first and then decreased. The soil organic matter content was the highest when the potassium application rate was K2, indicating that the appropriate application of potassium fertilizer could promote the increase of soil organic matter content, but excessive application of potassium fertilizer would lead to the decrease of organic matter content. Therefore, reasonable application of potassium fertilizer can help maintain and improve soil fertility. The results showed that with the increase of potassium application rate, the contents of organic matter and available potassium in the soil of *C. oleifera* forest increased significantly, while the contents of alkali-hydrolyzable nitrogen and available phosphorus in the soil decreased first and then increased (Luo et al., 2016). Studies have shown that the content of soil available phosphorus decreased with the increase of potassium application, while the content of soil nitrate nitrogen increased further. Under low and medium fertilization rates, soil nitrogen content was the most abundant and could be maintained for a long time, but high fertilization rate would lead to nitrogen leaching (Li et al., 2020). Therefore, appropriate amount of potassium application is beneficial to improve soil organic matter content and activate soil available nutrients. With the increase of potassium application rate, the total phosphorus content and available phosphorus content in the soil of *Q. tumidinoda* forest decreased first, and then increased when the potassium application rate reached 150 kg/hm^2^ (K2). This result indicated that the application rate of potassium fertilizer affected the absorption and accumulation of phosphorus in the clonal ramets of *Q. tumidinoda*, and when the potassium application rate reached 75 kg/hm^2^ (K1), the absorption of phosphorus in the clonal ramets of *Q. tumidinoda* had reached a saturated state (Luo et al., 2016). In summary, potassium fertilizer application can significantly improve soil nutrient status and promote plant nutrient uptake, but the effect is dose-dependent. Excessive application of potassium may lead to the inhibition of nutrient absorption. Therefore, to determine the optimal potassium application rate, it is necessary to comprehensively consider the soil background fertility, plant nutrient demand characteristics and the synergistic/antagonistic effects of other nutrient elements, so as to realize the efficient utilization of nutrient resources.

## 5 Conclusion

The application of potassium fertilizer can effectively improve the different quality of bamboo shoots. Soluble sugar content, starch content and protein content were significantly increased. The increase of crude fat content was not significant, but there was a significant decrease; the effect of crude fiber content was not significant, the content of essential amino acids increased significantly (except K4 treatment), the content of umami amino acids and the total amount of amino acids increased significantly. Appropriate amount of potassium fertilizer application can improve the comprehensive quality of bamboo shoots, but excessive fertilization will lead to the decline of the comprehensive quality of bamboo shoots. Appropriate application of potassium fertilizer significantly reduced the soil pH value and increased the content of soil organic matter, total phosphorus, total potassium and available nutrients. With the increase of potassium application rate, the contents of soil organic matter, total nitrogen and total potassium increased first and then decreased, while the content of total phosphorus increased continuously. In summary, the appropriate amount of potassium fertilizer can not only improve the nutritional quality of bamboo shoots, but also significantly improve the soil organic matter and nitrogen, phosphorus and potassium nutrient content. However, in order to achieve the best fertilization effect, it is necessary to apply balanced fertilization in combination with other elements.

## Data Availability

The raw data supporting the conclusions of this article will be made available by the authors, without undue reservation.
