# Supplementary figures and images for: Effects of potassium fertilizer application on Qiongzhuea tumidinod shoots nutritional quality and soil nutrients
Source: Front Plant Sci. 2025 Dec 10;16:1686259. doi: 10.3389/fpls.2025.1686259 (PMC12727894; doi:10.3389/fpls.2025.1686259)

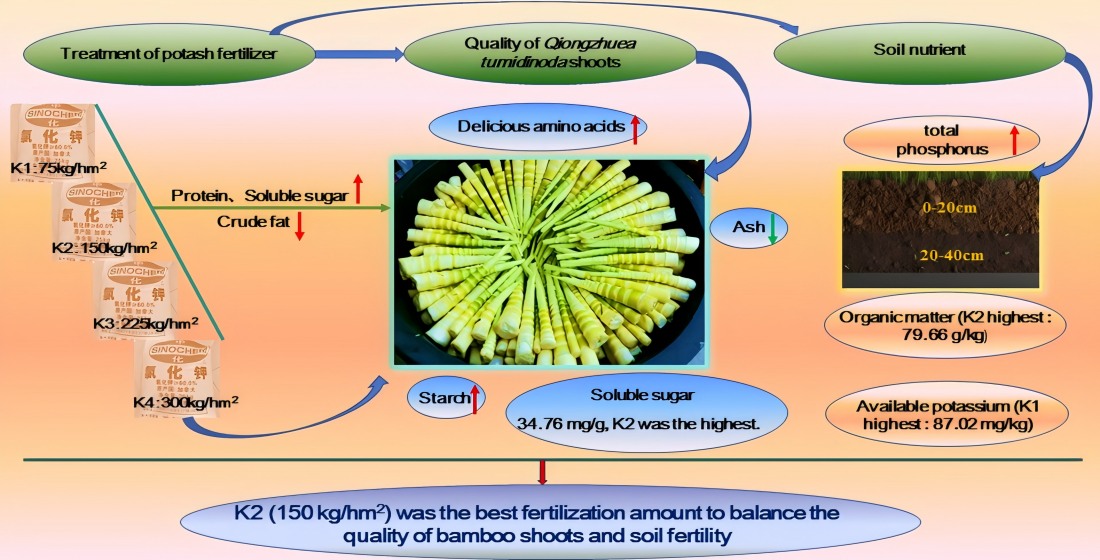

Supplement: Supplementary file 1 [file Image1.png]
